# Clinical research of genetically modified dendritic cells in combination with cytokine-induced killer cell treatment in advanced renal cancer

**DOI:** 10.1186/1471-2407-14-251

**Published:** 2014-04-10

**Authors:** Danhong Wang, Bin Zhang, Haiyan Gao, Guoliang Ding, Qiong Wu, Jinchao Zhang, Li Liao, Hu Chen

**Affiliations:** 1 Department of Hematopoietic Stem Cell Transplantation, Affiliated Hospital of Academy of Military Medical Sciences, Beijing, China; 2 Cell and Gene Therapy Center, Academy of Military Medical Sciences, Beijing, China

**Keywords:** Clinical research, Dendritic cells, Cytokine-Induced Killer cell, Advanced renal cancer

## Abstract

**Background:**

Renal cell carcinoma (RCC) is a malignant disease that demonstrates resistance to standard chemotherapeutic agents. Yet Active immunization using genetically modified dendritic cells holds promise for the adjuvant treatment of malignancies to eradicate or control residual disease. Cytokine-induced killer (CIK) cells are a heterogeneous population of effector CD8^+^ T cells with diverse TCR specificities, possessing non-MHC-restricted cytolytic activities against tumor cells. Clinical studies have confirmed benefit and safety of CIK cell-based therapy for patients with malignancies. This clinical trial was conducted to evaluate efficacy and safety of genetically modified dendritic cells in combination with Cytokine-Induced Killer Cell (gmDCs-CIK) treatment of patients with RCC.

**Methods:**

28 patients with advanced renal cancer were admitted to Affiliated Hospital of Academy of Military Medical Sciences from December 2010 to March 2012 and treated by gmDCs-CIK. Clinical efficacy and safety between pre- and post-treatment were compared.

**Results:**

This analysis showed an objective response rate (ORR) of 39% and a disease control rate (DCR) of as 75%. There is no significant relationship between clinical efficacy and whether metastasis occurred or not (P > 0.05). There is no significant relationship between ORR and cycles of treatment (P > 0.05), but DCR was significantly related with cycles of treatment (P < 0.05). No clinically significant side effects were observed. There were no significant changes of T cell subsets including CD3^+^, CD4^+^, CD8^+^, CD4^+^ CD25^+^ Treg cells except Th1 in peripheral blood between day 30 after immunotherapy and 1 day before immunotherapy in 11 patients.

**Conclusion:**

DC-CIK is feasible and effective in treating advanced renal cancer and thus provides a new approach.

**Trial registration:**

ClinicalTrials.gov Identifier: NCT01924156. Registration date: August 14, 2013.

## Background

Renal cell carcinoma (RCC) accounts for about [1] 5% of all new cancer cases worldwide. It is estimated that 27, 0000 new cases will be diagnosed with renal cancer in the world [2] and its incidence is rising each year. Radical nephrectomy can be curative for early stage disease, but for those patients with distant metastases the prognosis is poor. After complete resection of the primary tumor, recurrence develops in another 30% of patients [3]. RCC remains a therapeutic challenge because of its resistance to conventional therapies such as radiation, chemotherapy, and hormonal therapy**.** Although immunotherapy using interleukin-2 (IL-2) or interferon-alpha (IFN-α) [4] has become an accepted standard treatment for patients with RCC benefits, it was limited to a minority of patients. Therefore, attempts to develop more effective and nontoxic therapeutic strategies are needed. Dendritic cells (DCs) are professional antigen-presenting cells, as they are endowed with the unique potential to activate anti-tumor effector T and B lymphocytes [5]. They have been applied in clinics. The first study using DC vaccination for patients with RCC was published in 1999 and altogether 225 clinical trials have been published so far. Cytokine-induced killer (CIK) cells, which are non-major histocompatibility complex (MHC)-restricted CD3^+^CD56^+^T cells, take advantage of the body’s natural ability to eliminate tumor cells by stimulating and restoring the immune system to recognize and kill tumor cells. The first clinical study applying autologous CIK cells for cancer therapy was performed by Schmidt-Wolf and colleagues in 1999 [6].

Recently, clinical trials were performed aiming at combining active immune therapy using tumor vaccines with passive immunotherapy using CIK cells [7]. Evidence is rising that the application of CIK cells in combination with pulsed DC may indeed improve the immune response towards cancer. To improve therapeutic potency of CIK cells by vaccination, Sun et al. made use of DCs in combination with CIK cells for the treatment of relapsed or refractory non-Hodgkin’s lymphoma (NHL) [8]. After immunotherapy, the CD3^+^CD8^+^:CD3^+^ CD56^+^ T cell ratio was improved and IFN-gamma and IL-12 levels were higher in patients of the DCs–CIK group compared to the CIK group. Tumor volume was substantially decreased. Except of transient fever and chill, no remarkable adverse events happened during or after the treatment. Although a small number of patients were treated, data imply that DCs in combination with CIK cells exhibit an improved anti-tumor immune response.

In this study, we evaluated the efficacy and safety of genetically modified DCs in combination with CIK in patients with RCC.

## Methods

### Patient selection

The study protocol was approved by the Institutional Review Board of the Affiliated Hospital of Academy of Military Medical Sciences. Patients were informed of the investigative nature of this study, and written consent in accordance with institutional regulations was obtained prior to study entry. Eligibility criteria were histopathologically confirmed diagnosis of advanced renal cancer (stageIIIB-IV), age more than 18 years, performance status less than 2 [9] and expected survival duration of more than 3 months; Exclusion criteria were a history of autoimmune disease, evidence of active infection, seropositivity for HIV or hepatitis B surface antigen, use of immunosuppressive agents, or pregnancy. Patients were also excluded if chemotherapy or immunomodulatory treatment had been conducted during the previous 4 weeks.

### Generation of DCs and CIK cells

#### DCs Vaccine preparation

Peripheral blood mononuclear cells (PBMC) were isolated by Ficoll-Paque (PAN Biotech, Aidenbach, Germany) density gradient centrifugation that were seeded in 75 cm3 culture flasks at a density of 5 ×10^6^ cells/ml for 2 hours at 37°C in RPMI 1640 medium (Biowhittaker, Verviers, Belgium). After 2 hours, the non-adherent cells were removed for the purpose of CIK culturing while adherent mono-cytes were cultured for DCs in RPMI 1640 supplemented with 1000 units/ml granulocyte macrophage colony-stimulating factor (GM-CSF; Novartis, New Jersey, USA) and interleukin-4 (IL-4) 500 U/ml (Strathmann Biotech, Hannover, Germany) for 6 days. On day 4, DCs maturation could be achieved by adding 100 ng/ml tumor necrosis factor-α (TNF-α) (R&D Systems, Minneapolis, MN,USA); On day 6, DCs were pulsed with RNA encoding antigen muc-1 and survivin. On day 7, DCs were harvested, washed, and suspended again to a final concentration of 1–9 × 10^7^cells/ml in saline solution for injection.

### Generation of CIK

Non-adherent cells were prepared in CIK medium (RPMI 1640 medium containing 500 U/ml rhIL-2 and 100 ng/ml IFN-γ (Hofman La Roche), 50 ng/ml Anti-CD3 (e-Bioscience) at the density (3–5) × 10^6^/ml, and then were seeded in 2 L culture bag. Cells were incubated in a humidified atmosphere of 5% CO^2^ at 37°C. CIK medium was changed or added according to the proliferation. CIK cells were harvested on day 11 and 13.

### Phenotypic analysis

The phenotype of DCs and CIK was determined by flow cytometry using a FACSCalibur (Becton Dikinson, San Jose, CA, USA). Cell staining was performed using FITC-or PE-conjugated mouse antibodies against CD80^+^, CD86^+^,MHCII, CD83^+^ and CD14^+^ (Immunotech, Westbrook, ME, USA) for DCs, as well as CD3^+^, CD4^+^, CD8^+^CD56^+^ (PharMingen, San Diego, CA, USA) for CIK. For staining, 1 × 10^5^ DCs or CIK cells were suspended in 100 μl of PBS and were incubated with 10 μl of the antibodies for 30 minutes at 4°C.

### Immunization protocol

Patients received four subcutaneous injections of 2-5 × 10^7^ cells of Gene-Modified DCs at groin, axilla and neck on days 7, 9, 11, and 13 respectively; patients received i.v. infusion of 2-5 × 10^10^ CIK on both days 11 and 13. IL-2 (2 million IU/m^2^) was administered every other day during 2-weeks interval. Each cycle of immunotherapy consisted of 4 injections of DCs and 2 injections of CIK.

### Evaluation of T lymphocytes subset

For evaluation of the immune status of RCC patients, CD3^+^, CD4^+^, CD8^+^, CD4^+^/CD8^+^ ratio, CD4^+^ CD25^+^Treg cells and Th1/Th2 cell were determined by FCM from peripheral blood 1 days before immunotherapy and on day 30 after immunotherapy.

### Objectives

The primary objective of the study is to determine the feasibility and safety of gmDCs-CIK cell treatment in RCC. The secondary endpoints were the overall response rate (ORR) and disease control rate (DCR). Patients were assessed serially using computed tomography of chest, abdomen and technetium bone scan. Clinical responses to vaccination were evaluated according to the World Health Organization criteria. Complete response (CR) was defined as complete disappearance of all clinically detectable disease. Partial response (PR) was defined as ≥ 50% decrease in the sum of the products of the two longest perpendicular diameters of all measurable lesions without the appearance of new lesions. Stable disease (SD) was defined as 25% increase or 50% decrease in tumor size. Progressive disease (PD) was defined as ≥25% increase in existing lesions or the appearance of a new metastasis.

### Advert effects

Toxicities were graded according to National Cancer Institute Common Toxicity Criteria (NCI-CTC, version 2.0).

### Statistical methods

Relationship between metastatic status and ORR/DCR as well as treatment cycles and ORR/DCR were evaluated with Fisher’s exact test. Th1/Th2 and T cell subsets were analyzed with either paired-t tests or nonparametric Wilcoxon signed-rank tests based upon the normality of data. A value of p < 0.05 was considered significant. The SPSS 16.0 software package (SPSS, Inc.) was used.

## Results

### Patient characteristics and phenotypic analysis

From December 2010 through March 2012, 28 patients (mean age: 51.5 years; 23 men and 5 women) with metastatic RCC with clear cell histology were enrolled, with all of them had an ECOG performance status smaller than 1 upon initial screening. Of these patients, thirteen patients received one cycle of immunotherapy, seven received two cycles, five received three cycles, and two received four cycles. Demographics are summarized in Table 1.

**Table 1:** Patient characteristics and response

**Pt #**	**Gender**	**Histology**	**Metastatic disease site**	**Vax #**	**Clinical response before immunotherapy**	**Clinical response after immunotherapy**	**Additional treatment before immunotherapy**	**Adverse effects (grade)**	**Survival in months**
01	F	CC	Liver	1	PD	PD	Sunitinib	No	20 m+
02	M	CC	Lung, bone	1	PD	PD	None	No	20 m+
03	M	CC	Lung, bone	1	PD	PR	None	No	20 m+
04	M	CC	Lung, bone	2	PD	SD	None	No	19 m+
05	M	CC	Lung, Liver	6	SD	PR	None	No	19 m+
06	M	CC	Bone	4	PD	SD	None	No	18 m+
07	M	CC	Bone	1	PD	SD	None	No	17 m+
08	M	CC	Lung	4	SD	PR	None	No	18 m+
09	M	CC	Lung, Liver, Brain	2	PD	SD	None	No	21 m+
10	M	CC	Chest	3	PD	SD	None	No	16 m+
11	M	CC	Lung	1	PD	PR	None	No	16 m+
12	M	CC	Liver	1	PD	PD	None	Fever (grade1)	15 m+
13	M	CC	Neck lymph	1	PD	CR	None	No	10 m+
14	M	CC	Lung	1	PD	SD	None	No	10 m+
15	M	CC	Lung	3	PD	PR	None	No	9 m+
16	M	CC	Lung	3	PD	CR	None	No	10 m+
17	M	CC	Abdominal	1	PD	CR	None	No	15 m+
18	M	CC	Lung	1	PD	PD	None	No	12 m+
19	M	CC	peritoneum lymph	1	SD	PR	None	No	21 m+
20	F	CC	Liver, Bone	1	PD	PD	None	No	15 m+
21	F	CC	Abdominal	2	PD	PD	None	No	16 m+
22	M	CC	Bone	2	SD	CR	sorafenib	No	20 m+
23	M	CC	Lung, Bone	3	SD	SD	None	No	6 m+
24	F	CC	Lung, Bone	1	PD	death	None	No	4 m+
25	M	CC	Lung, Liver	3	PD	PR	Sorafenib + Sunitinib	No	6 m+
26	F	CC	Peritoneum lymph	2	SD	SD	None	No	11 m+
27	M	CC	Lung, Bone	2	PD	SD	None	No	5 m+
28	M	CC	Lung	2	SD	SD	sorafenib	No	5 m+

*Abbreviations*: *PT* patient, *VAX* vaccination, *M* male, *CC* renal clear cell carcinoma, *F* female, *SD* stable disease, *CR* completed response, *PR* partial response, *PD* progression of disease.

The mature DCs phenotype was reflected by a mean percent positive value of 5.7 ± 10.0 for monocyte marker CD14^+^, 60.4 ± 23.7% for DC marker CD83^+^, and 90.8 ± 13.0%, 83.2 ± 22.6%, and 81.7 ± 13.6% for MHCII, CD80^+^, and CD86^+^, respectively. Phenotypic analysis of CIK cells in 28 patients and after 11 days of culture showed that percentages of CD3^+^, CD3^+^CD4^+^, CD3^+^CD8^+^, CD3^+^CD56^+^ cell subsets is 82.06% ± 9.21%, 43.70% ± 6.08%, 36.31 ± 5.16%, and 18.24% ± 4.71%, respectively.

### Immunologic responses to gmDCs-CIK

**Figure 1:**
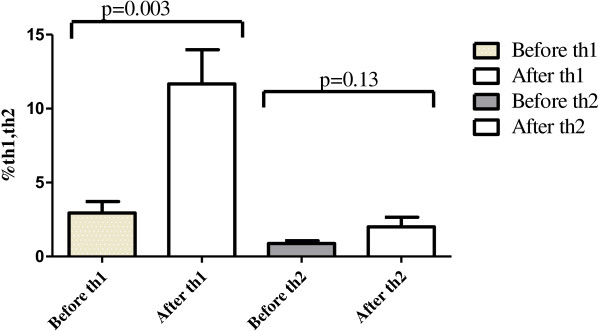
The changes of Th1/Th2 after treatment for 11 patients.

Peripheral blood lymphocyte subset proportions were measured using flow cytometry. The percentage of CD3^+^, CD3^+^CD4^+^, CD3^+^CD8^+^, CD16^+^CD56^+^ and CD4^+^CD25^+^ treg cell and CD4^+^/CD8^+^T cell ratio did not differ significantly between 1 day before immunotherapy and day 30 after immunotherapy (*P* > 0.05). There is significant difference for Th1 but not for Th2 (Figure 1) by flow cytometry.

### Clinical outcomes and safety

Among all those 28 patients who were available for clinical assessment, there are 4 CR with 2 of them more than 10 months and the other 2 more than 15 months, 7 PR (6.0-21.0 months), and 10 SD (5.0-21.0 months), 6 PD, and 1 death. ORR is 39% and DCR 75%. Follow-up ranges from 4 to 21 months.

During the immunotherapy, no adverse events with grade greater than 2 were reported. Patients with only flu-like symptoms with fever were noticed but didn’t require additional treatment (shown in Table 1).

### Association of ORR/DCR with other variables

**Figure 2:**
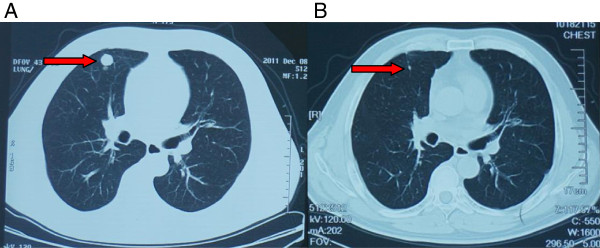
**Clinical response to gmDCs-CIK treatment patient with renal cancer.** CT scans of patient with renal cancer showing lung metastases. **(A)** arrow, sites are from lung metastases before gmDCs-CIK treatment, **(B)** arrow, sites are from lung metastases after gmDCs-CIK treatment.

Significant correlations were not found between ORR/DCR and metastatic site (p > 0.05) (Table 2). However, for patients with metastasis especially from lung site, there was great reduction in tumor size after immunotherapy (Figure 2). Correlations were not found between ORR and cycles of treatment, whereas correlations were found between DCR and cycles of treatment (i.e. 1 cycle vs. more than 1 cycle) (Table 3).

**Table 2:** The relationship between ORR and cycles of immunotherapy

**Cycle of treatment**	**Therapeutic effect (n)**		**Total**	**Effective rate (℅)**	**P value (Exact Sig. (2-sided)**
	**Effective**	**No effect**			
1	5	8	13	38.5	1.000
≥2	6	9	15	40	
Totle	11	17	28	39.3	

**Table 3:** The relationship between DCR and cycles of immunotherapy

**Cycle of treatment**	**Therapeutic effect (n)**		**Total**	**Effective rate (℅)**	**P value (Exact Sig. (2-sided)**
	**Effective**	**No effect**			
1	7	6	13	53.8	0.029*
≥2	14	1	15	93.3	
Total	21	7	28	75	

*P value less than 0.05 indicates statistical significance.

## Discussion

Treatment of RCC, especially metastatic RCCs, confronts a great dilemma in clinical practice. Although there are a number of therapeutic options available such as the immuno-regulating cytokines and the new anti-angiogenic targeted agents at present, these are commonly toxic and rarely produce durable complete remissions. The recent considerable success of cell immunotherapy in melanoma warrants further efforts to apply this treatment to other cancers including RCC.

Based on the antigen specificity of the immune system and the safety profile of cancer vaccines, the effective immunotherapy would be an ideal adjuvant, following initial clinical responses to definitive therapy [10]. In 2010, Yang B conducted autologous cytokine induced killer cells combined with IL-2 for therapy of elderly patients with B-cell malignant lymphoma [11]. The results showed that no adverse reaction was observed in all above mentioned patients. The percentages of CD3^+^,CD3^+^CD8^+^ and CD3^+^CD56^+^ increased significantly (p < 0.05), and serum levels of β2-microglobulin and LDH were markedly decreased (p < 0.05) after autologous CIK cell transfusion. The lymphoma symptoms were relieved with quality of life obviously elevated (p < 0.01) in all patients. Complete remission was seen in 8 patients. In conclusion, autologous CIK cells combined with IL-2 is safe and effective for the therapy of elderly patients with B-cell malignant lymphoma. In 2009, Thomas Schwaab and colleagues made use of autologous dendritic Cell with IL-2 therapy in RCC Patients [12]. The results showed all patients received between two and five treatment cycles. Overall objective clinical response rate was 50% with three complete responses. Treatment-related changes in correlative immunologic end points were noted.

On the basis of above research reports, we propose a new combinatorial therapy approach to mobilize anti-tumor immunity against RCC. Our results indicate that vaccination of these patients with gmDCs-CIK is safe and efficient. We found a ORR (39%) and a CRR (12%) which compared favorably to the historical observations of ORR (16%) and CR (6%) for high-dose IL-2 or IL-2 plus IFN-α therapy [13-16]. In addition, Turnbull JD and his colleages had reported results of a clinical trial [17] of bevacizumab in treating mRCC showing an ORR of 9.5% which was not superior to ORR in our study. Moreover, data of DCR is superior to other related clinical research [18,19]. For example, Kadono Y et al. had conducted a clinical trial of IFN-α in systemic treatment on 15 patients with mRCC enrolled from June 2005 through September 2008 [20] showing an ORR of 7% and a DCR of 27% which is lower than our DCR of 75%.

Clinical response of immunotherapy was evaluated by WHO and RESIST in a large number of clinical trial. However there is now ample evidence that these criteria do not apply to immunotherapy [21]. Immunotherapy induced tumor regressions have been well documented after initial progression and even after the appearance of new lesions, which are presumably caused by the infiltration of lymphocytes into tumours. For example, in some patients receiving ipilimumab, metastases may grow or new lesions may even develop before there is a decline in total tumor burden. These observations have led to the proposal of novel immune-related response criteria, as response evaluation according conventional response criteria (such as WHO and RECIST) can lead to unwanted early cessation of treatment owing to initial tumor growth [22]. These observations reflect the different dynamics of the immune response compared with the direct effects of cytotoxic drugs on cancer cells [23]. This also has important implications for the design and conduct of clinical trials, such as the planning of interim analyses.

Historically, cancer immune therapies have focused on stimulation of effector cells. In the present study, we observed no obvious difference between pre-vaccination and post-vaccination in Th2 but Th1 is significant higher than pre-vaccination (p = 0.003). Subsets of lymphocyte were evaluated in 11 patients with RCC treated with gmDCs-CIK. Result shows CD3^+^, CD4^+^, CD4^+^/CD8^+^,CD56^+^, CD4^+^CD25^+^doesn’t have significantly difference (P > 0.05) as compared with treatment before. Th cells are central to the development of an immune response by activating antigen-specific effector cells and recruiting cells of the innate immune system [24]. Two predominant Th cell subtypes exist, Th1 and Th2. Th1 cells, characterized by secretion of IFN-gamma and TNF-alpha, are primarily responsible for activating and regulating the development and persistence of CTL. In addition, Th1 cells activate antigen-presenting cells (APC) and induce limited production of the type of antibodies that can enhance the uptake of infected cells or tumor cells into APC. Th2 cells favor a predominantly humoral response. Th1 immune response is considered more effective than T helper 2 (Th2) for anti-tumor immunity. In this study, Th1 is significant higher than pre-vaccination by immunotherapy which indirectly proves that the immunological effect. Most data on lymphocyte subsets in malignant disease originate from melanoma or renal cell carcinoma (RCC) studies. There are several studies implying that the relative amount of CD3^+^, CD4^+^, CD8^+^, and CD56^+^ may be important and by reducing the tumor burden. However, only some of these studies imply that these changes can have an correlation on clinical outcome and prognosis [21]. Our results showed no correlation between recent clinical efficacy and metastatic sites from either bone or lung. This differed from results from other references for the difference of TNM staging of renal cancer, time of immune treatment, cycle of treatment, and time of testing of the peripheral blood lymphocyte subsets [25]. However, at present there is a lack of robust assays to monitor the anti-tumor immune response. Although there is an abundance of different assays that are being used to measure tumor antigen-specific T cell responses, these assays have not shown consistent results among trials, and none has been validated in prospective clinical trials.

## Conclusion

In conclusion, genetically modified DCs in combination with CIK represent a novel immunotherapy approach for treating advanced RCC. gmDCs-CIK is safe and effective for RCC. We have revealed, for the first time, the relationship between cycle of treatment and the DCR; our study also has indicated that immunotherapy could improve the clinical efficacy of advanced renal cancer and increased frequency of immunotherapy could result in additional benefits. A larger scale clinical trial should be conducted to confirm the conclusion. Standardization of immunotherapy needs to be the focus of ongoing research.

## Competing interests

The authors declare that they have no competing interests.

## Authors’ contributions

DW carried out all of the assays as well as the statistical analysis and she was helped by GD in some of the procedures, such as preparation of cell culture, and analyzing the data. HC conceived this study and designed in the experiments. BZ, HG, QW, JZ and LL provided technical and intellectual support, participating in the results and methodological discussions. All authors helped to draft this article and read and approved the final manuscript.

## Pre-publication history

The pre-publication history for this paper can be accessed here:

http://www.biomedcentral.com/1471-2407/14/251/prepub
